# Depressive symptoms among children attending community based support in South Africa – pathways for disrupting risk factors

**DOI:** 10.1177/1359104520935502

**Published:** 2020-06-23

**Authors:** Lorraine Sherr, Alexa R Yakubovich, Sarah Skeen, Mark Tomlinson, Lucie D Cluver, Kathryn J Roberts, Ana Macedo

**Affiliations:** 1 Institute for Global Health, University College London, UK; 2 Centre for Evidence-Based Intervention, Department of Social Policy & Social Intervention, University of Oxford, UK; 3 Department of Psychology, Stellenbosch University, South Africa; 4 Department of Psychiatry and Mental Health, University of Cape Town, South Africa

**Keywords:** Children, depressive symptomology, South Africa, LMIC, mental health

## Abstract

Children in Southern Africa are exposed to high rates of structural and family adversities. This study tests whether services from Community Based Organisations (CBOs) in South Africa can promote children’s resilience against depression exposed to such adversities. Two linked longitudinal studies were conducted, comprising *n* = 1848 children aged 9 to 13 years. One group received CBO services, whilst the other (quasi-control) did not. Analyses used interaction terms in regression models to test for potential moderation effects of CBO attendance, and marginal effects models to interpret significant interactions. Two interaction effects were shown, demonstrating moderation effects of CBO attendance on common structural disadvantages. First, children exposed to community violence showed increased depression (contrast = 0.62 [95%CI 0.43, 0.82], *p* < .001), but this association was removed by CBO access (contrast = 0.07 [95%CI −0.28, 0.43], *p* = .682). Second, children living in informal housing showed increased depression (contrast = 0.63 [95%CI 0.42, 0.85], *p* < .001), however, this association was removed by CBO access (contrast = 0.01 [95%CI −0.55, 0.56], *p* = .977). CBO attendance is associated with fewer depressive symptoms, and can buffer against important structural adversities of poor housing and violence that are common in high HIV-prevalence areas. However, CBO attendance was not able to remove the increased psychosocial distress associated with some family-level vulnerabilities such as orphanhood and abuse. These findings highlight the centrality of CBO-provided psychosocial support for children in Southern Africa, and suggest areas for bolstering provision.

## Introduction

The HIV/AIDS epidemic has exposed many children in Southern Africa to severe structural disadvantages that raise risks of mental health distress. Affected families experience substantial burdens of mortality and morbidity, parental and child illness, as well as a widespread psychological burden (Sherr et al., 2014; Stein et al., 2014). The transmission dynamics of the disease result in multiple burdens that cluster within families, with implications for developmental trajectories of children (Li et al., 2015). Parental illness and death are extreme consequences – with lifelong implications for child wellbeing (Cluver et al., 2013). In addition, children themselves may be infected or exposed to HIV in utero. Disease management, treatment side effects, and disease symptoms can affect parental availability, wellbeing, employment, and nurturing ability. Moreover, the burden of HIV/AIDS can further contribute to strains on children’s living environment, such as increased poverty, diversion of household assets to healthcare, and relationship disruption (Richter et al., 2014). HIV and AIDS-affected children also experience contribution demands such as replacement caregiving for others in the household and education disruptions due to lack of funds increased household responsibilities for caregiving or finance generation needs (Richter, 2010).

Exposure to violence – whether at home or in the community – also has negative impacts on children’s emotional health. High rates of violence and harsh punishment against children have been recorded in the African region (Hillis et al., 2016) and are exacerbated in households experiencing concurrent stresses such as illness, poverty, informal housing, overcrowding and community violence (Meinck et al., 2016).

There is often a mental health burden for children affected by HIV (Sherr et al., 2018; Skeen et al., 2016, 2017) including mood disorders (depression, suicidal behaviours and anxiety), trauma (posttraumatic stress), psychosocial challenges (including stigma, social withdrawal, poor confidence, poor self-efficacy and poor self-esteem; Gentz et al., 2018; Mellins et al., 2018; Vreeman et al., 2017; Walsh et al., 2017). Many of the lived experiences in HIV-affected households in resource limited settings, such as the communities from which the data for this study was obtained, can contribute to child difficulties (Collishaw et al., 2016; Gamarel et al., 2017; Woollett et al., 2017). For example, children exposed to household overcrowding, unemployment, orphanhood, abuse, community violence, and informal housing are known to be at greater risk for depression (Anda et al., 2006; Barbarin et al., 2001; Cluver & Gardner, 2007; Cluver et al., 2015; Gilman et al., 2002). Yet, despite exposure to many structural challenges, there is sound evidence that some children show resilience (Betancourt et al., 2013; Masten et al., 2014; Skovdal et al., 2012). What remains unclear is whether such resilience is an individual characteristic, or whether community support – or an alternative intervention – can foster and build resilience behaviours at best, or at least, shelter children from the more extreme effects of such exposure. In particular, resilience is theorized as a dynamic interactive process between a child, their family, and external supports (Luthar & Cicchetti, 2000; Yates et al., 2003), with increasing focus on the ‘social and physical ecology’ of community-based support in low-resource settings (Ungar, 2011).

Community-based organisations (CBOs) may be an important form of mental health provision for children in low-resource contexts with generalised HIV epidemics – both as a response to trauma and as a preventive intervention that can build resilience (Strebel, 2004; Williamson, 2000). Community-based organisations have historically been an important intervention strategy for addressing these unique psychosocial needs of high risk children generally and HIV-affected children specifically (Sherr et al., 2016, Strebel, 2004; Williamson, 2000). In theory, CBOs are low-cost, adaptive to community needs, locally accessible, and capable of providing programming for vulnerable children and families (Foster, 2007; Yakubovich et al., 2016). In the absence of formal health infrastructure and training, a number of community-based services have evolved to provide for such needs. These may include home visits, parenting or early child education, social support, counselling, referrals, financial assistance and healthcare provision.

However, evaluations of the psychosocial effects of real-world CBOs (as opposed to those that have only been set up for the purposes of research) among HIV-affected children have been limited, largely as a result of factors such as low research capacity, inadequate funding, and small programme sizes (King et al., 2009; Schenk, 2009; Skeen et al., 2017). As the new Sustainable Development Goals underscore the importance of wellbeing – a step beyond the more basic survival goals of the Millennium Development goals – it is timely to consider the potential protective effects of CBO provision on child wellbeing.

Bolstering the hypothesis that CBOs may serve as a protective factor for child wellbeing, we previously found that CBOs were successfully reaching children who were in many ways more vulnerable than those not being reached, (Sherr et al., 2016; Yakubovich et al., 2016). Specifically, using data combined from two longitudinal studies of children in South Africa, which faces the world’s largest generalised HIV epidemic (UNAIDS, 2016), we found that children attending CBOs tended to live in largely unemployed or overcrowded households, be orphaned, have high care-giving responsibilities, and have been exposed to more community violence. Yet, despite these vulnerabilities, children attending CBOs had better psychological wellbeing, measured as fewer depressive symptoms, both at initial interview and 1-year follow-up (Sherr et al., 2016; Yakubovich et al., 2016). These studies thus raise an important further question; whether attending CBOs contributes to building psychological resilience – namely strengthening capacity to do well despite multiple adversities – among vulnerable children affected by HIV/AIDS.

These analyses were therefore set up to explore whether attending a CBO was, in fact, *moderating* the relationship between common family and structural vulnerabilities and child depressive symptoms. We hypothesised that by providing services that address the psychosocial needs of vulnerable children and their families, CBOs may have a buffering effect on children exposed to socio-demographic vulnerabilities experienced in economically disadvantaged communities in South Africa, who would otherwise be expected to be at greater risk for depression. That is, CBOs may not be able to change children’s exposure to all of these vulnerabilities, particularly those that are at the structural-level such as community violence and poor housing conditions. However, by providing psychosocial support, these organisations may be able to protect children from the otherwise severe negative mental health consequences these exposures can cause. This hypothesis would be supported if the family and structural level factors available in our sample were indeed related to having more depressive symptoms among children who did not attend CBOs, but showed a weaker (or null) association with depressive symptoms among children attending CBOs.

## Method

### Participants and procedure

Data were drawn from two South African longitudinal studies, conducted over similar time periods with a series of common measures. The Child Community Care study (CCC) provided data on children who regularly attended CBOs, while the Young Carer (YC) study served as a quasi-control group, where child data was available with the specific inclusion criteria for non-attendance and no links with CBO provision at all.

*Child Community Care Study*: The CCC is a study of children aged 4 to 13 affected by HIV/AIDS and receiving services from CBOs in two Southern African countries: South Africa and Malawi. The combined database used in the current study is confined to data from South Africa only for the purpose of matching with YC data. Measured used for matching are detailed in the measures section below. Twenty-four South African CBOs were randomly sampled from a list of 588 CBOs supported by 11 funding partners, stratified by funder and geographical region. All CBOs provided direct services to children. Table 1 provides an overview of the services provided by the CBOs and an overview of the proportion of children who accessed each service. From the CBOs selected, approximately 35 children were consecutively sampled from each CBO. The refusal rate at baseline interviews (2011–2012) was low (<1%) 1% and retention at follow-up was high (86%; 12–15 months later). Participants completed a 60-min face-to-face interview with a trained data collector using mobile phone technology (Tomlinson et al., 2009) at two time points. Baseline data was collected 2011 to 2012 and follow-up data was collected 12 to 15 months later. Questionnaires were translated and back-translated in Zulu and Xhosa and children participated in the language of their choice.

**Table 1.** Types of community based programme services children in the child community care (CCC) study received at baseline based on caregiver report.

Characteristic of the organisation provided	Accessed	No. children accessing CBO services (*n* = 446)
Food and nutrition services	Yes	258 (57.8%)
	No	188 (42.2%)
Medical services	Yes	30 (6.7%)
	No	416 (93.3%
Play services	Yes	248 (55.6%)
	No	198 (44.4%)
Early child development programming	Yes	100 (22.4%)
	No	346 (77.6%)
Education services	Yes	127 (28.5%)
	No	319 (71.5%)
Emotional support services	Yes	73 (16.4%)
	No	373 (83.6%)
Home based care services	Yes	121 (27.1%)
	No	325 (72.9%)
Social grant access assistance	Yes	63 (14.1%)
	No	383 (85.9%)
Training and skills building provided	Yes	37 (8.3%)
	No	409 (91.7%)

*Young Carers Study*: YC participants were randomly selected from two urban and two rural health districts with over 30% antenatal HIV prevalence in two South African provinces (Mpumalanga and the Western Cape). Sampling involved randomly selecting census enumeration areas from the four health districts, visiting every household in the selected areas, and randomly selecting one child from every household with a resident aged 9 to 18 years. Similar to CCC, refusal was low (<2.5%). Participants were interviewed at baseline (2009–2010) and 1-year follow-up (2011–2012) with 96.8% retention. As in CCC, participants completed a 60-min face-to-face interview with trained data collectors and questionnaires were translated and back-translated in Xhosa, Zulu, Sotho, and Shangaan, with children participating in the language of their choice.

*Creating the combined database*: The sample for the analyses reported in this study was obtained from the databases of YC and CCC, focusing on the overlapping age range of 9 to 13 years between the two studies. Only data from South African participants were used. YC questionnaires included detailed measures of CBO provision, which were used to establish a subgroup of children from YC who had no exposure at either baseline or follow-up to any form of CBO service. For CCC, there were 446 children eligible for inclusion in the combined database (drawn from South Africa and aged from 9–13 years). For YC, there were 1,402 children eligible for inclusion. The total sample for the current paper was thus 1,848 South African children aged 9 to 13 years at baseline, with 107 children lost to follow-up. Differences between children retained at and lost to follow-up are discussed below.

*Ethical procedures*: YC ethical protocols received approval from the Universities of Oxford, Cape Town, and KwaZulu-Natal, and provincial Health and Education Departments. CCC ethical protocols were approved by University College London (reference number 1478/002) and Stellenbosch University (reference number N10/04/112) and the funding agencies supporting the sampled CBOs. In the CCC, voluntary written consent was provided by caregivers and verbal assent by children. In YC, both caregivers and children provided voluntary written consent to participate. Participants of both studies did not receive incentives apart from refreshments, food, and certificates of participation. Confidentiality was maintained in both studies, except when participants were at risk of significant harm or requested assistance. In such cases, immediate referrals were made to local health and social services and/ or partnering CBOs.

### Measures

For the current analyses, we chose variables within our combined database that used identical measures in both the YC and CCC studies. All measures described below were self-reported by child participants. Type of services for the CCC participants were reported by caregivers.

*Socio-demographics*: Age and gender were measured using national census items (Statistics South Africa, 2001). (In)formal housing was measured by having participants indicate in which of different types of houses they lived (i.e. a house/flat, a shack, or on the street). Orphanhood was defined in accordance with UNAIDS as the loss of one or both biological parents. Physical abuse was measured with two items and defined as carers using a stick/belt to hit the child or slapping/punching the child at least weekly (Snider & Dawes, 2006). Household size and employment were measured in both studies by having participants count how many people live in their home and the employment status of each. Exposure to community violence was measured using two binary (yes/no) items of the Child Exposure to Community Violence Checklist, having (a) seen someone being attacked and (b) personally been attacked outside the home (Richters & Martinez, 1993). Answering ‘yes’ to either of these was defined as exposure to community violence.

*Outcome*: Depressive symptomology was measured using a short-form of the Children’s Depression Inventory (nine items, scored 0–2; Kovacs, 1992). This is a well-established standardised measure of childhood depression that has been used in an African and South African context. Scores were summed for a total depressive score, with higher scores indicating a greater number of depressive symptoms (α = .67).

### Analyses strategy

A five-stage analyses strategy was conducted in Stata 13.2. First, the sample was described on all variables at both time points. Second, we regressed depressive symptoms at follow-up onto a ‘full model’, including all baseline risk factors and their interactions with CBO attendance vs. non-attendance, controlling for baseline depressive symptoms, age, and gender. Third, we removed interaction terms which were not statistically significant at *p* < .10 and re-ran this ‘reduced model’. Fourth, we tested whether the reduced model significantly impaired goodness of fit using the likelihood-ratio test. Fifth, we computed average marginal effects to interpret each statistically significant interaction term.

## Results

### Differences between participants lost to and retained at follow-up

Relevant differences between participants lost and retained at follow-up in the total sample, CCC sub-sample, and YC sub-sample are summarised in Table 2, including difference statistics and *p*-values for each comparison. Although the retention rate in this combined sample was extremely high (approximately 94%), there were some differences between those who were and were not retained. Briefly, no statistically significant differences were found on: gender, informal housing, household employment, physical abuse, or depressive symptoms. However, children who were lost to follow-up were on average 3 months younger than those retained (*M* = 11.21 years, *SD* = 1.2 and *M* = 11.47 years, *SD* = 1.2, respectively), more often orphans (41.5%) compared to those retained at follow-up (30.0%), and more often living in informal housing (22.2%) compared to those retained (12.0%). Violence and abuse rates were lower among children lost to follow-up compared to those retained: Fewer children lost to follow-up had experienced regular emotional abuse (2.8% vs 8.0%), or seen someone be attacked on the street (25.2% vs 36.3%). The latter difference was driven by YC participants, among whom those lost to follow-up had been exposed to violence less often (0.0%) compared to those who were retained at follow-up (34.5%). Where participants lost-to-follow-up were more vulnerable at baseline, results may underestimate risk; where they were less vulnerable, results may overestimate risk.

**Table 2.** Baseline comparison between participants who were lost and retained at follow-up.

	Lost to follow-up			Retained at follow-up			Difference statistic (***p***-value)
	Total, ***n* = 107**	CCC, ***n* = 63**	YC, ***n* = 44**	Total, ***n* = 1741**	CCC, ***n* = 383**	YC, ***n* = 1358**	
**Depressive symptoms**	1.41 (2.1)	1.19 (1.8)	1.73 (2.4)	1.09 (1.8)	0.77 (1.2)	1.17 (1.9)	1.832 (.067)
**Female gender**	54 (50.5%)	28 (44.4%)	26 (59.1%)	957 (55.0%)	197 (51.4%)	760 (56.0%)	0.824 (.364)
**Age**	**11.21 (1.2)**	10.89 (1.3)	11.68 (1.1)	**11.47 (1.2)**	10.95 (1.3)	11.62 (1.1)	**2.137 (.033)**
**Orphan (at least one parent died)**	**44 (41.5%)**	34 (54.8%)	10 (22.7%)	**520 (30.0%)**	235 (62.5%)	285 (21.0%)	**6.238 (.013)**
**Weekly physical abuse**	5 (4.7%)	1 (1.6%)	4 (9.1%))	92 (5.3%)	1 (0.3%)	91 (6.7%)	0.076 (.783)
**Weekly emotional abuse**	**3 (2.8%)**	0 (0.0%)	3 (6.8%)	**139 (8.0%)**	10 (2.6%)	129 (9.5%)	**3.813 (.051)**
**Seen someone be attacked**	**27 (25.2%)**	27 (42.9%)	0 (0.0%)***	**631 (36.3%)**	163 (42.7%)	468 (34.5%)***	**5.348 (.021)**
**⩾1 Employed person in the household**	68 (63.6%)	36 (57.1%)	32 (72.7%)	1250 (71.8%)	213 (55.6%)	1037 (76.4%)	3.388 (.066)
**Informal housing**	32 (29.9%)	14 (22.2%)*	18 (40.9%)	453 (26.0%)	46 (12.0%)*	407 (30.0%)	0.787 (.375)

Data are mean (SD) or *n* (%). Difference statistic is chi-square for categorical variables or t-score for continuous variables. The difference statistic indicates the statistical significance of the difference between *total* retained for follow-up and *total* lost to follow-up on each variable (i.e. across both studies). Statistically significant differences are bolded. Asterisks indicate the statistical significance of differences between participants retained and lost at follow-up *for each study* (i.e. separately for CCC participants and YC participants), where *p < .05, **p < .01, and ***p < .001.

### Full effects model examining depression over time

The unstandardised B coefficients, 95% confidence intervals, and p-values for the full effects model are presented in Table 3. The interaction terms that were statistically significant (defined in this stage as *p* < .10) were CBO attendance by (a) exposure to community violence and (b) informal housing. These interaction terms were thus retained for the reduced model and all other interaction terms removed.

**Table 3.** Full effects model examining depression over time.

Variable	B	[95% CI]	*p*-value
Constant	0.913	–	–
CBO attender	−0.101	[−0.703, 0.603]	.880
**Child factors**			
Depressive symptoms	0.140	[0.092, 0.188]	<.001
Female gender	0.074	[−0.091, 0.239]	.382
Age	−0.006	[−0.077, 0.065]	.875
**Family factors**			
Orphan	0.018	[−0.212, 0.247]	.880
Orphan × CBO attender	−0.101	[−0.534, 0.332]	.647
Weekly physical abuse	0.111	[−0.270, 0.493]	.567
Physical abuse × CBO attender	2.388	[−1.081, 5.857]	.177
Household size	−0.064	[−0.110, −0.019]	.006
Household size × CBO attender	0.056	[−0.023, 0.135]	.165
**Structural factors**			
Exposure to community violence	0.619	[0.423, 0.814]	<.001
Community violence × CBO attender	−0.559	[−0.965, −0.154]	.007
Household unemployment	−0.212	[−0.431, 0.007]	.058
Household unemployment × CBO attender	−0.047	[−0.476, 0.381]	.828
Informal housing	0.619	[0.404, 0.833]	<.001
Informal housing × CBO attender	−0.615	[−1.222, −0.007]	.047

*n* = 1731.

### Reduced model for depressive symptoms at follow-up

The unstandardised B coefficients, 95% confidence intervals, and *p*-values for the reduced model are presented in Table 4. The reduced model did not impair goodness-of-fit compared to the full model, χ^2^ = 4.31, *p* = .366, and was therefore kept as the final analysis model for the sake of parsimony.

**Table 4.** Reduced model examining depression over time.

Variable	B	[95% CI]	*p*-value
Constant	0.829	–	–
CBO attender	0.241	[−0.075, 0.530]	.140
**Child factors**			
Depressive symptoms	0.141	[0.093, 0.189]	<.001
Female gender	0.063	[−0.102, 0.228]	.455
Age	−0.008	[−0.079, 0.063]	.825
**Family factors**			
Orphan	−0.006	[−0.200, 0.188]	.951
Weekly physical abuse	0.167	[−0.234, 0.524]	.453
Household size	−0.045	[−0.082, −0.008]	.017
**Structural factors**			
Exposure to community violence	0.623	[0.427, 0.818]	<.001
Community violence × CBO attender	−0.549	[−0.952, −0.146]	.008
Household unemployment	−0.228	[−0.415, −0.040]	.018
Informal housing	0.635	[0.422, 0.847]	<.001
Informal housing × CBO attender	−0.627	[−1.214, −0.039]	.037

*n* = 1731.

As expected, children with more depressive symptoms at initial interview had more depressive symptoms at follow-up, controlling for all other variables in the model, *B* = 0.14 [95% CI 0.09, 0.19]. In contrast, children who were living in larger households or without any employed person had fewer depressive symptoms at follow-up, *B* = −0.05 [95% CI −0.08, −0.01] and *B* = −0.23 [95% CI −0.42, −0.04].

The interaction terms between CBO attendance and each of exposure to community violence and informal housing remained statistically significant, *p* = .008 and *p* = .037, respectively. These interaction terms are interpreted below.

### Interpreting interactions between CBO attendance and structural disadvantage using average marginal effects

We computed average marginal effects for each statistically significant interaction term – specifically, CBO attendance by (a) community violence and (b) informal housing – across all other variables in the reduced model (Table 4). The contrast value referred to in what follows indicates the discrete difference in the adjusted mean value of depressive symptoms between participants (a) exposed versus not exposed to community violence or (b) living in informal versus formal housing. Positive contrast values indicate that participants exposed to community violence or living in informal housing at initial interview had more depressive symptoms at follow-up than those not exposed to community violence or living in informal housing, respectively. Whether the 95% confidence interval includes 0 indicates whether this difference was statistically significant at α = .05.

**Figure 1.**
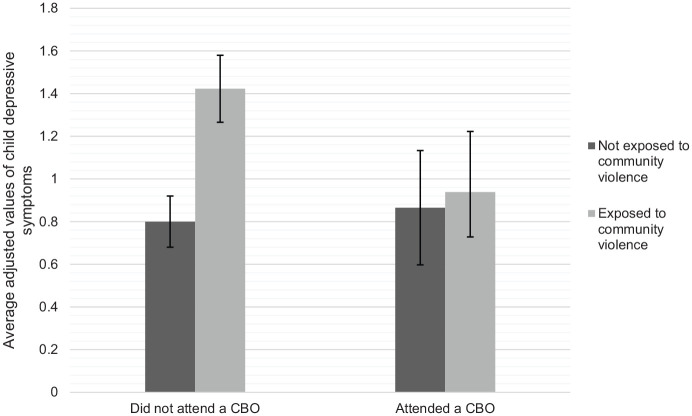
The average adjusted values of depressive symptoms among participants exposed and not exposed to community violence by CBO attendance. Adjusted values are averaged across all levels of the variables shown in Table 4. Error bars indicate the 95% confidence interval of the average adjusted value.

**Figure 2.**
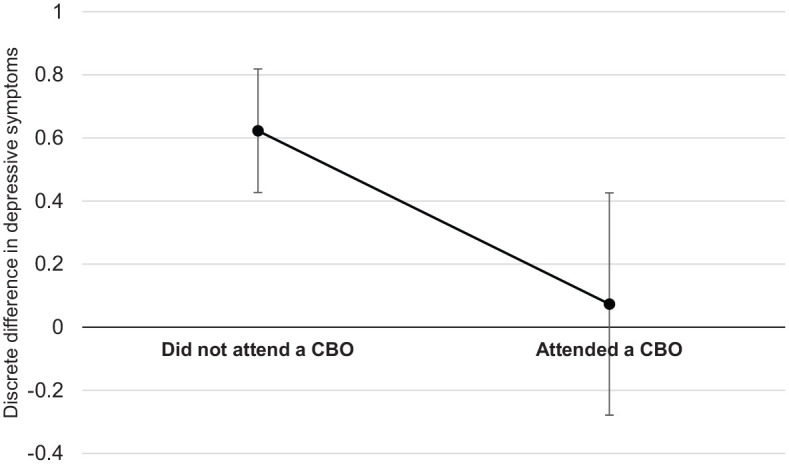
The discrete difference between the adjusted mean values of depressive symptoms among participants exposed versus not exposed to community violence by CBO attendance. Model controls for all variables shown in Table 4. Positive discrete difference scores indicate that the adjusted mean of depressive symptoms was higher among participants exposed to community violence compared to those not exposed. Error bars indicate the 95% confidence interval of the difference. The bolded x-axis indicates the line of no effect (i.e. when difference = 0).

As shown in Figure 1, when children were not accessing CBO services, the adjusted mean value of the number of self-reported depressive symptoms (averaged across all other variables in the model, see Table 4) was much higher among children exposed to community violence (*M* = 1.42, 95% CI 1.27, 1.58) than those not exposed to this violence (*M* = 0.80, 95% CI 0.68, 0.92). In contrast, when children were regularly accessing CBO services, the adjusted mean values of depressive symptoms were similar among children with exposure to community violence (*M* = 0.94, 95% CI 0.65, 1.22) and without this exposure (*M* = 0.87, 95% CI 0.60, 1.13).The statistical significance of these differences in the adjusted means of depressive symptoms among children exposed versus not exposed to community violence and attending versus not attending CBOs are summarised in Figure 2. The contrast value referred to in what follows indicates the discrete difference in the adjusted mean value of depressive symptoms between participants (a) exposed versus not exposed to community violence or (b) living in informal versus formal housing. Positive contrast values indicate that participants exposed to community violence or living in informal housing at initial interview had more depressive symptoms at follow-up than those not exposed to community violence or living in informal housing, respectively. Whether the 95% confidence interval includes 0 indicates whether this difference was statistically significant at α = .05. As can be seen in Figure 2, among participants who did not attend CBOs, exposure to community violence at initial interview was associated with having more depressive symptoms at follow-up, contrast = 0.62 [95% CI 0.43, 0.82], *p* < .001. Whereas among participants who did attend CBOs, exposure to community violence was not associated with depressive symptoms, contrast = 0.07 [95% CI −0.28, 0.43], *p* = .682.

**Figure 3.**
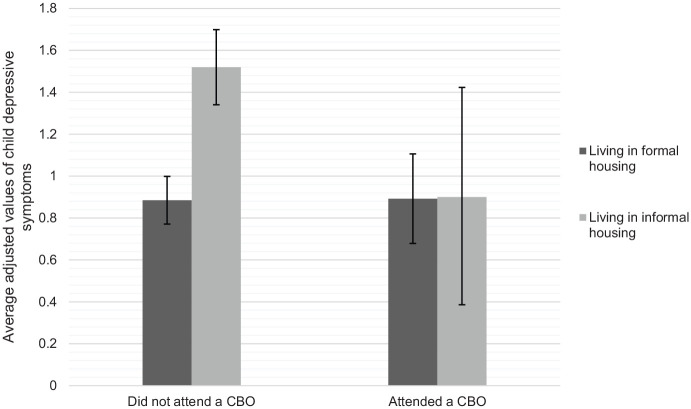
The average adjusted values of depressive symptoms among participants living in informal and formal housing by CBO attendance. Adjusted values are averaged across all levels of the variables shown in Table 4. Error bars indicate the 95% confidence interval of the average adjusted value.

**Figure 4.**
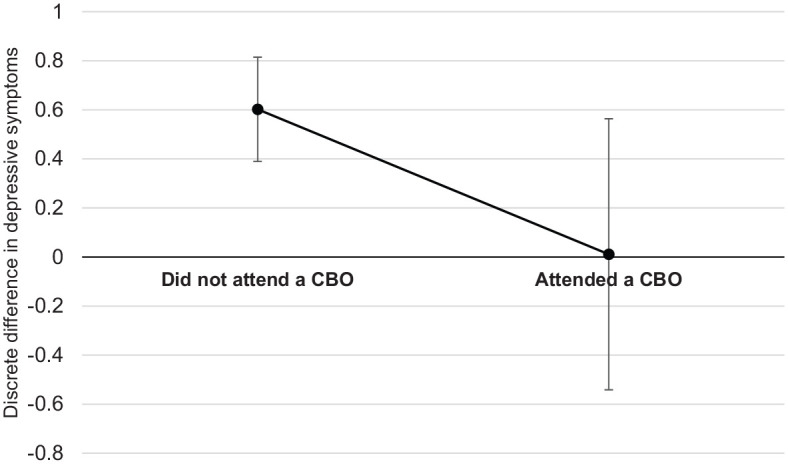
The discrete difference between the adjusted mean values of depressive symptoms among participants living in informal versus formal housing by CBO attendance. Model controls for all variables shown in Table 4. Positive discrete difference scores indicate that the adjusted mean of depressive symptoms was higher among participants living in informal housing compared to those living in formal housing. Error bars indicate the 95% confidence interval of the difference. The bolded x-axis indicates the line of no effect (i.e. when difference = 0).

A similar pattern was observed in the depressive symptoms of children living in informal versus formal housing who were receiving versus not receiving CBO services. The adjusted mean values of the number of self-reported depressive symptoms by informal housing and CBO attendance (adjusted for all variables in Table 4) are shown in Figure 3. When children were not accessing CBO services, the adjusted mean value of the number of depressive symptoms was much higher among children living in informal housing (*M* = 1.52, 95% CI 1.34, 1.70) as compared to those living in formal housing (*M* = 0.89, 95% CI 0.77, 1.00). In contrast, when children were regularly accessing CBO services, the adjusted mean values of depressive symptoms were similar among children living in informal (*M* = 0.90, 95% CI 0.38, 1.42) and formal housing (*M* = 0.89, 95% CI 0.70, 1.11).The significance test of these differences in the average adjusted depressive symptoms of participants are summarised in Figure 4. As can be seen, among participants who did not attend CBOs, those living in informal housing at initial interview had more depressive symptoms at follow-up than those living in formal housing, contrast = 0.63 [95% CI 0.42, 0.85], *p* < .001. In contrast, there was no association between living in informal housing and depressive symptoms at follow-up among CBO-attending participants, contrast = 0.01 [95% CI −0.55, 0.56], *p* = .977.

## Discussion

This study investigated whether CBO attendance moderates the relationship between a range of family and structural vulnerabilities and child depressive symptoms. In particular, we examined whether three family-level factors – orphanhood, physical abuse, and household size – as well as three structural factors – exposure to community violence, household unemployment, and living in informal housing – are differentially associated with depressive symptoms among children depending on whether or not they are receiving CBO services.

Our findings suggest that CBO attendance moderated the association between two structural-level factors at initial interview (exposure to community violence and living in informal housing) and depressive symptoms at 1-year follow-up. That is, children who were not attending CBOs had higher levels of depressive symptoms when they were exposed to community violence or living in informal housing as compared to when they were not. In contrast, among children attending CBOs, exposure to community violence and living in informal housing was not associated with the severity of depressive symptoms, suggesting that CBO support may protect against the mental health risks associated with these disadvantages.

There are several possible pathways that may explain such results. First, the relationships between child depressive symptomology and the exposure to community violence and informal housing may both reflect ‘neighbourhood effects’. A large body of evidence in the last 30 years has demonstrated that characteristics of neighbourhoods or communities are important determinants of poor health and behaviour (Diez Roux & Mair, 2010; Macintyre et al., 2002). Although historically this evidence base focused on physical health and criminal behaviour, increasingly, research has demonstrated the effects of the community environment on depression (Cutrona et al., 2006; Kim, 2008). Physical features of the environment such as poor-quality housing may increase daily life stresses, which in turn increase the risk for child depressive symptomology (Evans et al., 2003). Informal housing may also be a proxy for lower socioeconomic status (and therefore fewer available resources), decreased security, and higher residential mobility, all of which can increase psychological distress and depressive symptoms among children. Moreover, greater exposure to violence in their communities increases the fear and threat of victimisation, which may then lead to more severe depressive symptoms among children and adolescents (McDonald & Richmond, 2008).

In turn, there are a number of ways in which CBOs may play a role in altering or deflecting the system of such ‘risk mechanisms’ of informal housing and exposure to community violence. Potentially this may be done by directly providing psychosocial services, acting as a source of stability, offering space for children to play safely (King et al., 2009; Skeen et al.,2017), fostering and enabling resilience through shared experiences and opportunities to learn coping mechanisms, or providing access to practical help or social resources to ameliorate the intensity of exposure to multiple community risks. In addition, by providing a combination of economic resources and parenting programmes, CBO provision may serve to buffer against the psychological risks for children associated with living in informal housing and more violent community environments by strengthening family environments, enhancing good parenting, reducing harsh punishment and allowing for school attendance (Richter et al., 2009; Sherr et al., 2016; Yakubovich et al., 2016). Alternatively, it may be possible that those families who are accessing CBOs may be more motivated to seek support and thus, this factor may play a part in mitigating depressive symptoms in the sample.

Yet the study did not find moderating effects of CBO attendance on the associations between depressive symptoms and all the family-level stresses investigated. As such, CBO services may simply not be sufficient or appropriate for a range of other community-level vulnerabilities such as overcrowded households, abusive environments or unemployment. It is unclear whether there were no effects on these conditions because of the type of provision or the quantity of provision. Not surprisingly, CBO attendance did not moderate the association between orphanhood and depressive symptoms. This suggests that the order of emotional impact from such catastrophic life events, such as the death of a parent, is not well served by CBO provision. In such instances, either the CBOs need to have enhanced skills to manage the death of a caregiver, or have the facility to screen and refer to other services – if such services exist. It may also be the case that the lack of protective effects observed were driven by weaker associations between these family-level stresses and higher depressive symptoms among children in the current sample (regardless of CBO attendance). Indeed, household unemployment and size had unexpected positive associations with child depressive symptoms, suggesting further exploration may be warranted.

There are a number of limitations in the current study which should be considered. The data on CBO attenders and non-attenders were obtained from two separate sources and thus differences observed between these two groups of children could potentially be the result of differences in methodology. However, this study only used measures that were identical between the two data sources to minimise the effects of potential measurement differences. In addition, all research assistants for both original studies were similarly recruited, trained and used similar interview techniques. A second limitation is that the data analysis was designed post-hoc and confined to the available variables with common measures. A prospectively-designed study to confirm our findings would be advised as the next step. Moreover, as this study measured real-world CBO attendance, this could not be randomized and children were not interviewed prior to attending these organisations: therefore conclusions regarding causality cannot be made. For instance, it is possible that CBO attendance is associated with another variable that is instead responsible for the moderated effects we observed, highlighting the need for replication. Finally, the data are limited to one country and to a 4-year age range, although there was a wide geographical spread within South Africa and this is an important developmental stage for child mental health.

HIV/AIDS continues to directly and indirectly affect millions of children in resource-limited contexts, placing their health and psychological wellbeing at risk. CBOs may offer a unique opportunity to provide local services that can protect and improve the mental health of children in areas of high HIV prevalence, despite the structural vulnerabilities they may face. Prospective data are needed to confirm the positive effects of CBOs on child mental health. Nevertheless, the current study adds to an encouraging evidence base that shows that not only are CBOs associated with fewer depressive symptoms among children, but these organisations may buffer against the psychological risks of more intransigent structural conditions like community violence and informal housing. CBOs remain a promising intervention strategy for vulnerable children and families and their impacts deserve further, rigorous investigation.

## References

[bibr1-1359104520935502] Anda R. F. Felitti V. J. Bremner J. D. Walker J. D. Whitfield C. Perry B. D. Dube S. R. Giles W. H. (2006). The enduring effects of abuse and related adverse experiences in childhood. A convergence of evidence from neurobiology and epidemiology. European Archives of Psychiatry and Clinical Neuroscience, 256(3), 174–186. 10.1007/s00406-005-0624-4 16311898 PMC3232061

[bibr2-1359104520935502] Barbarin O. A. Richter L. M. de Wet T. (2001). Exposure to violence, coping resources, and psychologicla adjustment of South African children. American Journal of Orthopsychiatry, 71(1), 16–25. 11271713 10.1037/0002-9432.71.1.16 PMC1866189

[bibr3-1359104520935502] Betancourt T. S. Meyers-Ohki S. E. Charrow A. Hansen N (2013). Annual research review: Mental health and resilience in HIV/AIDS-affected children—A review of the literature and recommendations for future research. Journal of Child Psychology and Psychiatry, 54(4), 423–444. 10.1111/j.1469-7610.2012.02613.x 22943414 PMC3656822

[bibr4-1359104520935502] Cluver L. D. Gardner F. (2007). Risk and protective factors for psychological well-being of children orphaned by AIDS in Cape Town: A qualitative study of children and caregivers’ perspectives. AIDS Care, 19(3), 318–325. 17453564 10.1080/09540120600986578

[bibr5-1359104520935502] Cluver L. D. Orkin F. M. Boyes M. E. Sherr L. (2015). Child and adolescent suicide attempts, suicidal behavior, and adverse childhood experiences in South Africa: A prospective study. Journal of Adolescent Health, 57(1), 52–59. 10.1016/j.jadohealth.2015.03.001 25936843

[bibr6-1359104520935502] Cluver L. Orkin M. Boyes M. E. Sherr L. Makasi D. Nikelo J. (2013) Pathways from parental AIDS to child psychological, educational and sexual risk: Developing an empirically-based interactive theoretical model. Social Science & Medicine, 87, 185–93. 10.1016/j.socscimed.2013.03.028 23631794

[bibr7-1359104520935502] Collishaw S. Gardner F. Lawrence Aber J. Cluver L. (2016) Predictors of mental health resilience in children who have been parentally bereaved by AIDS in Urban South Africa. Journal of Abnormal Child Psychology, 44(4), 719–730. 26329481 10.1007/s10802-015-0068-x

[bibr8-1359104520935502] Cutrona C. E. Wallace G. Wesner K. A. (2006). Neighborhood characteristics and depression: An examination of stress processes. Current Directions in Psychological Science, 15(4), 188–192. 10.1111/j.1467-8721.2006.00433.x 18185846 PMC2186297

[bibr9-1359104520935502] Diez Roux A. V. Mair C (2010). Neighborhoods and health. Annals of the New York Academy of Sciences, 1186(1), 125–145. 10.1111/j.1749-6632.2009.05333.x 20201871

[bibr10-1359104520935502] Evans G. W. Wells N. M. Moch A. (2003). Housing and mental health: A review of the evidence and a methodological and conceptual critique. Journal of Social Issues, 59(3), 475–500.

[bibr11-1359104520935502] Foster G. (2007). Under the radar: Community safety nets for AIDS-affected households in sub-Saharan Africa. AIDS Care, 19(Suppl. 1), S54–S63. 10.1080/09540120601114469 17364388

[bibr12-1359104520935502] Gamarel K. E. Kuo C. Boyes M. E. Cluver L. D. (2017). The dyadic effects of HIV stigma on the mental health of children and their parents in South Africa. Journal of HIV AIDS Social and Service, 16(4), 351–366. 10.1080/15381501.2017.1320619 PMC5724576 29238272

[bibr13-1359104520935502] Gentz S. G. Calonge-Romano I. Martínez-Arias R. Zeng C. Ruiz-Casares M. (2018). Mental health among adolescents living with HIV in Namibia: The role of poverty, orphanhood and social support. AIDS Care, 30(Suppl. 2), 83–91 29848003 10.1080/09540121.2018.1469727

[bibr14-1359104520935502] Gilman S. E. Kawachi I. Fitzmaurice G. M. Buka S. L. (2002). Socioeconomic status in childhood and the lifetime risk of major depression. International Journal of Epidemiology, 31(2), 359–367. 11980797

[bibr15-1359104520935502] Gonzalez-Guarda R. M. Williams J. R. Williams W. Lorenzo D. Carrington C. (2019). Determinants of HIV and sexually transmitted infection testing and acquisition among female victims of intimate partner violence. Journal of Interpersonal Violence, 0886260519827662. 10.1177/0886260519827662 PMC6692236 30755076

[bibr16-1359104520935502] Hillis S. Mercy J. Amobi A. Kress H. (2016). Global prevalence of past-year violence against children: A systematic review and minimum estimates. Pediatrics, 137(3), e20154079. 26810785 10.1542/peds.2015-4079 PMC6496958

[bibr17-1359104520935502] Kim D. (2008). Blues from the neighborhood? Neighborhood characteristics and depression. Epidemiologic Reviews, 30(1), 101–117. 10.1093/epirev/mxn009 18753674

[bibr18-1359104520935502] King E. De Silva M. Stein A. Patel V. (2009). Interventions for improving the psychosocial well-being of children affected by HIV and AIDS. Cochrane Database of Systematic Reviews, 2, CD006733 10.1002/14651858.CD006733.pub2 PMC7387107 19370650

[bibr19-1359104520935502] Kovacs M. (1992). Children’s Depression Inventory. Multi-Health Systems.

[bibr20-1359104520935502] Li X. Chi P. Sherr L. Cluver L. Stanton B. (2015). Psychological resilience among children affected by parental HIV/AIDS: A conceptual framework. Health Psychology and Behavioral Medicine, 3(1), 217–235. 26716068 10.1080/21642850.2015.1068698 PMC4691449

[bibr21-1359104520935502] Luthar S. S. Cicchetti D. (2000). The construct of resilience: Implications for interventions and social policies. Development and Psychopathology, 12(4), 857–885. 11202047 10.1017/s0954579400004156 PMC1903337

[bibr22-1359104520935502] Macintyre S. Ellaway A. Cummins S. (2002). Place effects on health: How can we conceptualise, operationalise and measure them? Social Science & Medicine, 55(1), 125–139. 12137182 10.1016/s0277-9536(01)00214-3

[bibr23-1359104520935502] Masten A. S. (2014). Global perspectives on resilience in children and youth. Child development, 85(1), 6–20. 24341286 10.1111/cdev.12205

[bibr24-1359104520935502] McDonald C. Richmond T. R. (2008). The Relationship between community violence exposure and mental health symptoms in urban adolescents. Journal of Psychiatric and Mental Health Nursing, 15(10), 833–849. 10.1111/j.1365-2850.2008.01321.x 19012675 PMC2821658

[bibr25-1359104520935502] Meinck F. Cluver L. D. Boyes M. E. Loening-Voysey H. (2016). Physical, emotional and sexual adolescent abuse victimisation in South Africa: Prevalence, incidence, perpetrators and locations. Journal of Epidemiology and Community Health, 70(9), 910–916. 26962202 10.1136/jech-2015-205860 PMC5013157

[bibr26-1359104520935502] Mellins C. A. Xu Q. Nestadt D. F. Knox J. Kauchali S. Arpadi S. Kvalsvig J. Shrout P. E. Davidson L. L. (2018). Screening for mental health among young south African children: The use of the Strengths and Difficulties Questionnaire (SDQ). Global Social Welfare, 5(1), 29–38. 30038880 PMC6054470

[bibr27-1359104520935502] Richter L (2010). An introduction to family-centred services for children affected by HIV and AIDS. Journal of Internal AIDS Society, 13(Suppl. 2), S1 10.1186/1758-2652-13-S2-S1 PMC2890970 20573283

[bibr28-1359104520935502] Richter L. M. Mofenson L. M. (2014) Children born into families affected by HIV. AIDS, (Suppl. 3), S241–S244. 24991894 10.1097/QAD.0000000000000361

[bibr29-1359104520935502] Richter L. M. Sherr L. Adato M. Belsey M. Chandan U. Desmond C. Drimie S. Haour-Knipe M. Hosegood V. Kimou J. Madhavan S. Mathambo V. Wakhweya A. (2009). Strengthening families to support children affected by HIV and AIDS. AIDS Care, 21(Suppl. 1), 3–12. 10.1080/09540120902923121 22380973 PMC2903779

[bibr30-1359104520935502] Richters J. Martinez P. (1993). The NIMH community violence project: I. Children as victims of and witnesses to violence. Psychiatry, 56(1), 7–21. 8488215 10.1080/00332747.1993.11024617

[bibr31-1359104520935502] Schenk K. D. (2009). Community interventions providing care and support to orphans and vulnerable children: A review of evaluation evidence. AIDS Care, 21(7), 918–942. 20024749 10.1080/09540120802537831

[bibr32-1359104520935502] Sherr L. Cluver L. D. Betancourt T. S. Kellerman S. E. Richter L. M. Desmond C (2014). Evidence of impact: Health, psychological and social effects of adult HIV on children. AIDS, 28 (Suppl. 3), S251–S259. 10.1097/QAD.0000000000000327 24991898

[bibr33-1359104520935502] Sherr L. Hensels I. S. Tomlinson M. Skeen S. Macedo A. (2018) Cognitive and physical development in HIV-positive children in South Africa and Malawi: A community-based follow-up comparison study. Child Care Health and Development, 44(1), 89–98. 10.1111/cch.12533 PMC6086496 29047149

[bibr34-1359104520935502] Sherr L. Yakubovich A. R. Skeen S. Cluver L. D. Hensels I. S. Macedo A. Tomlinson M. (2016). How effective is help on the doorstep? A longitudinal evaluation of ccommunity-based organisation support. PLoS ONE [Electronic Resource], 11(3), e0151305. 10.1371/journal.pone.0151305 PMC4788449 26967732

[bibr35-1359104520935502] Skeen S. Macedo A. Tomlinson M. Hensels I. S. Sherr L. (2016). Exposure to violence and psychological well-being over time in children affected by HIV/AIDS in South Africa and Malawi. AIDS Care, 28(Suppl. 1), 16–25. 10.1080/09540121.2016.1146219 PMC4828604 27002770

[bibr36-1359104520935502] Skeen S. Sherr L. Tomlinson M. Croome N. Ghandi N. Roberts J. K. Macedo A. (2017). Interventions to improve psychosocial well-being for children affected by HIV and AIDS: A systematic review. Vulnerable Children and Youth Studies, 12(2), 91–116 29085436 10.1080/17450128.2016.1276656 PMC5659734

[bibr37-1359104520935502] Skovdal M. (2012). Pathologising healthy children? A review of the literature exploring the mental health of HIV-affected children in sub-Saharan Africa. Transcultural Psychiatry, 49(3–4), 461–491. 23008352 10.1177/1363461512448325

[bibr38-1359104520935502] Snider L. M. Dawes A. (2006). Psychosocial vulnerability and resilience measures for national-level monitoring of orphans and other vulnerable children: Recommendations for revision of the UNICEF Psychological Indicator. UNICEF.

[bibr39-1359104520935502] Statistics South Africa. (2001). Census 2001. Statistics SA.

[bibr40-1359104520935502] Stein A. Desmond C. Garbarino J. Van IJzendoorn M. H. Barbarin O. Black M. M. Stein A. D. Hillis S. D. Kalichman S. C. Mercy J. A. Bakermans-Kranenburg M. J. Rapa E. Saul J. R. Dobrova-Krol N. A. Richter L. M. (2014) Predicting long-term outcomes for children affected by HIV and AIDS: Perspectives from the scientific study of children’s development. AIDS, 28(Suppl. 3), S261–S268. 10.1097/QAD.0000000000000328 PMC10875626 24991899

[bibr41-1359104520935502] Strebel A. (2004). The development, implementation, and evaluation of interventions for the care of orphans and vulnerable children in Botswana, South Africa, and Zimbabwe: A literature review of evidence-based interventions for home-based child-centred development. Human Sciences Research Council.

[bibr42-1359104520935502] Tomlinson M. Solomon W. Singh Y. Doherty T. Chopra M. Ijumba P. Tsai A. C. Jackson D. (2009). The use of mobile phones as a data collection tool: A report from a household survey in South Africa. BMC Medical Informatics and Decision Making, 9(1), 51. 20030813 10.1186/1472-6947-9-51 PMC2811102

[bibr43-1359104520935502] UNAIDS. (2016). Global AIDS update. UNAIDS.

[bibr44-1359104520935502] Ungar M. (2011). The social ecology of resilience: Addressing contextual and cultural ambiguity of a nascent construct. American Journal of Orthopsychiatry, 81(1), 1–17. 10.1111/j.1939-0025.2010.01067.x 21219271

[bibr45-1359104520935502] Vreeman R. C. McCoy B. M. Lee S. (2017) Mental health challenges among adolescents living with HIV. Journal of International AIDS Society, 20(Suppl. 3), 21497 10.7448/IAS.20.4.21497 PMC5577712 28530045

[bibr46-1359104520935502] Walsh A. S. J. Wesley K. L. Tan S. Y. Lynn C. O’Leary K. Wang Y. Nguyen D. Chenneville T. Rodriguez C. A. (2017). Screening for depression among youth with HIV in an integrated care setting. AIDS Care, 29(7), 851–857 28278567 10.1080/09540121.2017.1281878

[bibr47-1359104520935502] Williamson J. (2000). Finding a way forward: Principles and strategies to reduce the impact of AIDS on children and families. Displaced Children and Orphans Fund and War Victims Fund Contract.

[bibr48-1359104520935502] Woollett N. Cluver L. Bandeira M. Brahmbhatt H. (2017) Identifying risks for mental health problems in HIV positive adolescents accessing HIV treatment in Johannesburg. Journal of Child Adolescent Mental Health, 29(1), 11–26. 28287023 10.2989/17280583.2017.1283320

[bibr49-1359104520935502] Yakubovich A. R. Sherr L. Cluver L. D. Skeen S. Hensels I. S. Macedo A. Tomlinson M. (2016). Community-based organizations for vulnerable children in South Africa: Reach, psychosocial correlates, and potential mechanisms. Children and Youth Services Review, 62, 58–64. 10.1016/j.childyouth.2016.01.016 27867244 PMC5113730

[bibr50-1359104520935502] Yates T. M. Egeland B. Sroufe L. A. (2003). Rethinking resilience: A developmental process perspective. In Luthar S. S. (Ed.), Resilience and vulnerability: Adaptation in the context of childhood adversities (pp. 243–266). Cambridge University Press.

